# Beverage Consumption Habits among the European Population: Association with Total Water and Energy Intakes

**DOI:** 10.3390/nu9040383

**Published:** 2017-04-13

**Authors:** Mariela Nissensohn, Almudena Sánchez-Villegas, Pilar Galan, Aida Turrini, Nathalie Arnault, Lorenza Mistura, Adriana Ortiz-Andrellucchi, Fabien Szabo de Edelenyi, Laura D’Addezio, Lluis Serra-Majem

**Affiliations:** 1 International Chair for Advanced Studies on Hydration (ICASH), University of Las Palmas de Gran Canaria, 35016 Las Palmas, Spain; marienis67@hotmail.com (M.N.); aortiza55@hotmail.com (A.O.-A.); 2 Research Institute of Biomedical and Health Sciences, University of Las Palmas de Gran Canaria, 35016 Las Palmas, Spain; almudena.sanchez@ulpgc.es; 3 CIBER OBN, Biomedical Research Networking Center for Physiopathology of Obesity and Nutrition, Carlos III Health Institute, 28029 Madrid, Spain; 4 Equipe de Recherche en Epidémiologie Nutritionnelle, Centre de Recherche en Epidémiologie et Statistiques, Université Paris 13, Inserm (U1153), Inra (U1125), Cnam, COMUE Sorbonne Paris Cité, F-93017 Bobigny, France; p.galan@uren.smbh.univ-paris13.fr (P.G.); n.arnault@uren.smbh.univ-paris13.fr (N.A.); f.szabo@uren.smbh.univ-paris13.fr (F.S.d.E.); 5 CREA-Consiglio per la ricerca in agricoltura e l’analisi dell’economia agraria–Centro di ricerca per gli alimenti e la nutrizione, Via Ardeatina 546, 00178 Rome, Italy; aida.turrini@crea.gov.it (A.T.); lorenza.mistura@crea.gov.it (L.M.); laura.daddezio@crea.gov.it (L.D.)

**Keywords:** total water intake, beverages, adults, France, Italy, Spain

## Abstract

Background: Fluid and water intake have received limited attention in epidemiological studies. The aim of this study was to compare the average daily consumption of foods and beverages in adults of selective samples of the European Union (EU) population in order to understand the contribution of these to the total water intake (TWI), evaluate if the EU adult population consumes adequate amounts of total water (TW) according to the current guidelines, and to illustrate the real water intake in Europe. Methods: Three national European dietary surveys have been selected: Spain used the Anthropometry, Intake, and Energy Balance Study (ANIBES) population database, Italy analyzed data from the Italian National Food Consumption Survey (INRAN-SCAI 2005-06), and French data came from the NutriNet-Santé database. Mean daily consumption was used to compare between individuals. TWI was compared with European Food Safety Authority (EFSA) reference values for adult men and women. Results: On average, in Spain, TWI was 1.7 L (SE 22.9) for men and 1.6 L (SE 19.4) for women; Italy recorded 1.7 L (SE 16.9) for men and 1.7 L (SE 14.1) for women; and France recorded 2.3 L (SE 4.7) for men and 2.1 L (SE 2.4) for women. With the exception of women in France, neither men nor women consumed sufficient amounts of water according to EFSA reference values. Conclusions: This study highlights the need to formulate appropriate health and nutrition policies to increase TWI in the EU population. The future of beverage intake assessment requires the use of new instruments, techniques, and the application of the new available technologies.

## 1. Introduction

Fluid and water intake have received limited attention in epidemiological studies. This hampers attempts to assess the adequacy of water intakes at the population level [1,2].

It is well known that adequate hydration status is associated with the preservation of physical and mental functions and that water intake is the best way to achieve hydration [3,4]. However, we have to be aware that there are other sources of liquids with similar hydration capacities, liquids with different flavors that also provide nutrients or stimulants, feed us, or are just more palatable, like milk, juices, teas, soups, beer and wine. During the last few decades, multiple types of drinks with different characteristics have been developed. Some of them are not only to satiate thirst. Soft drinks, flavored waters, or different kinds of infusions with different properties such as sedatives, digestives, antioxidants, etc. are some examples. The current selection of beverages is so broad that it is clear that there should be specific recommendations with respect to these liquids; this should include their capacity to hydrate and supply energy or other nutrients, as well as any other effects they may have on the body [5]. With this in mind, some recent studies have suggested that the variety of beverages consumed is a positive predictor of total water intake (TWI) [1,6,7,8].

On the other hand, in recent years, we have witnessed the emergence of obesity as a serious problem in the Western world. In fact, excessive consumption of sugary beverages has been linked to increasing weight of populations [9,10]. Unfortunately, there is little evidence to show that replacing caloric drinks with water has beneficial effects on body weight or sensitivity to insulin [11], although different studies have shown that replacing these drinks with water results in a decrease in total caloric intake [1,12]. Therefore, it seems to be prudent to encourage the consumption of drinking water instead of other caloric drinks [5].

Some countries and public organizations have proposed water intake recommendations for the general public [13,14,15,16]. They recognize that the value of adequate intake (AI) is a variable event, in which differences are due, in part, to the inter-individual variation for water needs in response to different health status, metabolism, and environmental factors, such as ambient temperature and humidity. Other individual factors, such as age, body size, and level of physical activity are involved [17]. Furthermore, water needs also depend on overall diet and the water contained in food. The European Food Safety Authority (EFSA) proposed Dietary Reference Values (DRV) for the Adequate Intake of Water per day. It included water from food and water from beverages. The range of DRVs in liters increase with age until 2.5 L and 2.0 L for 14+ year-old men and women, respectively [13].

Following the analyses of some European Union (EU) nutritional databases, the aim of this study was to compare the average daily consumption of fluids (water and other beverages) in selected samples of the EU populations in order to understand the contribution of each fluid type to total water intake. Furthermore, we will evaluate if the adult EU population consumes adequate amounts of total water according to EFSA [13] recommendations, or if those populations reached AI values defined as the ratio between TWI (g of water from food and beverages) and energy intake (EI) in kcal. This ratio suggests that water intake is inadequate when the result is less than 1. We will also explore associations between the types of beverage consumed and energy intake from the diet.

## 2. Materials and Methods

Three countries of the EU and their dietary surveys have been selected. Only adults ranging from 18 to 75 years were considered for the present study. The countries were chosen because of geographic proximity, similar climate and cultural characteristics, and by the method used to collect information of the food and beverage intake. Only those countries that used food records or 24-h dietary recalls were included. Food and beverage intake was recorded in each study by age and sex, as well as day and time of consumption.

### 2.1. Spain (ANIBES Dataset)

In Spain, the population database used was from the Anthropometry, Intake and Energy Balance Study (ANIBES), a cross-sectional study conducted using stratified multistage sampling. The representative sample of this national survey of diet and nutrition comprised 2285 healthy participants, aged 9–75 years. The sample was collected from the following geographical locations: Northeast, Levant, Southwest, North–Central, Barcelona, Madrid, Balearic, and Canary Islands. The fieldwork for the ANIBES study was conducted from mid-September, 2013, to mid-November, 2013, with the participation of 90 interviewers allocated in 11 areas and 12 coordinators, all previously trained by the Spanish Nutrition Foundation (FEN). The survey was performed “door-to-door” following randomized routes. For better results at the main fieldwork, different informative posters about ANIBES’ goals were posted in the area/neighborhood, followed by letters that were sent to all the neighbors. In addition, during the first visit by the interviewer, an informative letter from the principal investigator plus a leaflet and a set of infographics explaining the whole process were offered. Finally, the potential participant was informed about a small incentive (30 euros) for participation and a detailed final report including anthropometric data, physical activity level, and dietary/nutritional status, with an estimated value of 40–50 euros. Demographic variables collected included gender, unemployment rate, physical activity level assessed by the International Physical Activity Questionnaire (IPAQ) [18], and education level. Participants’ weight, height, and waist circumference were measured, and body mass index was also calculated. Study participants were provided with a tablet device (Samsung Galaxy Tab 2 7.0, Samsung Electronics, Suwon, South Korea). If the participant declared or demonstrated that he/she was unable to use the tablet device, other possibilities were offered: photo camera plus paper or telephone interview. Participants were trained to record information by taking photos of all food and drinks consumed during three consecutive days, both at home and outside of the home. To equally represent all days of the week, study subjects participated during two weekdays and one day during the weekend. Food records were returned from the field in real-time (i.e., at the exact moment in which they were consumed), to be coded by trained coders who were supervised by dieticians. Food, beverage, energy, and nutrient intakes were calculated from food consumption records using VD-FEN 2.1 software, a Dietary Evaluation Program from the Spanish Nutrition Foundation, Spain. This software was newly developed for the ANIBES study by the FEN and is based mainly on Spanish food composition tables [19], with several expansions and updates. A food photographic atlas was used to assist in assigning gram weights to portion sizes. Details of the ANIBES study have been described in detail elsewhere [1].

### 2.2. Italy (INRAN-SCAI Dataset)

Data from Italy come from the national food consumption survey (INRAN-SCAI 2005-06). This cross-sectional survey was conducted on a representative sample of 1300 randomly selected households between October 2005 and December 2006. Census data were used for the multistage stratification of the sample into the four main geographical areas of Italy (North-West, North-East, Centre, South, and Islands), provinces’ population size (large, medium, and small), municipalities’ population size (large-medium, and small), and four strata according to household composition. In each municipality, households were randomly selected from the telephone guide. In total, 3323 (1501 males and 1822 females) individuals participated in the food survey, aged 0 to 97 years. A three-day semi-structured diary was used for participants to record the consumption of all foods, beverages, and nutritional supplements. A team of trained field workers conducted the survey, and individually met the participants who self-recorded all foods and beverages consumed, estimating portion sizes with the help of a picture booklet. Food data were coded by field workers during the data entry using a data management system developed for the purpose of the survey. Other variables collected were age, gender, education, occupation, lifestyle (smoking, physical activity, dieting). Height and weight were self-reported. Data on nutrient intake—including water—were obtained using the updated version of the Italian national food composition database [20]. The “Food Energy and Nutrient Composition Database” was used to estimate the data on energy, macro-nutrients, dietary fiber, vitamins, and minerals. Detailed information about the INRAN SCAI 2005-06 survey design, procedures, and methodologies can be found in previously published papers [7,20,21].

### 2.3. France (NutriNet-Santé Dataset)

French data come from the NutriNet-Santé database, a web-based observational prospective cohort including volunteers aged 18 years or older, launched in France in May 2009, with a scheduled follow up in 10 years. The study was conducted on a large sample of 94,939 participants. It aimed at determining the association of food intake, nutrients, and dietary behavior with ageing and quality of life. At baseline, socio-demographic data including age, gender, education, income, occupational category, and household location, as well as lifestyle (smoking status, physical activity), height, weight, and practice of restrictive dieting were self-reported. Leisure-time physical activity was assessed using the French short form of the International Physical Activity Questionnaire, self-administered online [22,23,24]. Body mass index (BMI) was assessed using self-reported height and weight. Dietary data were collected at baseline using three-day records, randomly distributed within a two-week period, including two weekdays and one day of the weekend. Participants reported all foods and beverages consumed throughout the day: breakfast, lunch, dinner, and all other occasions. Daily mean food and beverage consumptions were calculated for each participant having completed the three-day records, with a weighting on the type of day (week or weekend) so that all days of the week were equally represented. Serving sizes were estimated using purchase units, household units, and photographs, and were derived from a previously validated picture booklet. EI and TWI were calculated through the NutriNet-Santé food composition table including more than 2000 food products [25]. The NutriNet-Santé study has been described in detail elsewhere [8,26].

### 2.4. Statistical Analysis

Eight different categories of beverages were used to examine beverage consumption and EI for each of the three studied countries: (1) hot beverages, including hot tea and coffee; (2) milk (all types of milk without separation by fat percentage); (3) fruit and vegetable juices (including nectars, juice–milk blends, 100% fruit juices); (4) caloric soft drinks (including colas; tonic water; sodas; ginger ale; fruit-flavored drinks; iced teas in cans or bottles; sports drinks, such as isotonic drinks with mineral salts; and caffeinated energy drinks); (5) diet soft drinks (including the same beverages as in the caloric soft drinks group but with artificial sweetener); (6) alcoholic drinks, including both low-alcohol grade and high-alcohol grade groups; (7) water (including tap water and bottled water); and (8) other beverages (including soy-based beverages, non-alcoholic beer and wine, and others).

All analyses were carried out separately in men and women and across countries. Differences in demographic and anthropometric variables between Spain, France, and Italy were assessed through chi-squared test for qualitative variables and through ANOVA-test when quantitative variables were compared. The analyses were focused on the TWI of all foods and beverages by country. Description of the population and the mean daily consumption of the food and beverage intakes were calculated for the 18–75-year-old population of each country. Contributions of food and beverages to TWI and EI were calculated. In each country, pairwise comparisons of the means across groups were assessed using *t*-test. A two-sided *p*-value of 0.05 was set to denote statistical significance.

TWI was compared with the EFSA [13] Dietary Reference Values for the Adequate Intake of water for men and women, from 14 years of age onward (2.5 L and 2.0 L, respectively). Furthermore, the ratio g/kcal was applied to provide a more comprehensive estimate of the proportion from each country that fulfilled the AI recommendations. This criterion is based on the water intake per unit of energy consumed. The value suggested is 1.0 L per 1000 kcal of EI [14]. However, this value could be increased to 1.5 L/1000 kcal depending on activity level and water loss. TWI for adults should be no less than 1.0 L/1000 kcal [13]. Therefore, we used three different approaches to define water intake adequacy in order to provide a more comprehensive estimate of the proportion of participants who consume low amounts of water. The first criterion is a classification based on the AI value, defined by the EFSA as Criterion 1. The second (Criterion 2) is a ratio between TWI (water from food and beverages in grams) and EI in kcal higher than 1. The combination of both is the final criterion (Criterion 3).

## 3. Results

Descriptive characteristics of the three included studies are presented in Table 1. The population of the studies ranged from 18 to 75 years. The studies included 1859 participants from Spain, 94,939 from France and 2313 from Italy. There were significant differences in most demographic and anthropometric variables between countries. The rate of unemployment shows that Spain had the highest percentage of unemployed participants—data that are in line with those from the statistical office of the European Union (Eurostat) [27]. For level of education, France’s males and females showed the highest University educated level.

The contribution of food and beverages to daily water intake (g/day) and EI (kcal/day) by genders are presented in Table 2. On average, in Spain, TWI for adults was 1.7 L (SE 22.9) for men and 1.6 L (SE 19.4) for women; Italy recorded 1.7 L (SE 16.9) for men and 1.7 L (SE 14.1) for women; and France recorded 2.3 L (SE 4.7) for men and 2.1 L (SE 2.4) for women. The mean daily EI for adults in Spain was 1790.8 kcal/day (SE 11.6), of which 12% was provided by beverages. For the Italian study, EI was 2137.9 (SE 12.2), and only 6% was supplied by beverages. France recorded an EI of 1884.5 (SE 1.5), of which 8% was provided by beverages.

**Figure 1:**
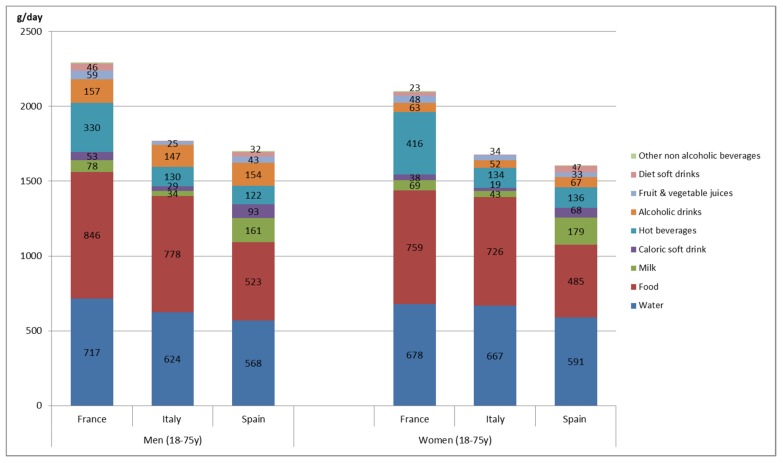
Daily water intake (g/day) provided by each beverage/food category according to gender among the populations included. All results are presented in g of water content in each category, not mean intakes by volume (e.g., g of water in milk, not g of milk consumed).

Figure 1 provides information on the amount (in grams) of water content in food and beverages consumed by country and gender. All results are presented in g of water content, not mean intakes by volume (e.g., g of water in milk, not g of milk consumed). Water was the most consumed beverage in the three countries for both sexes, followed by the water provided by milk in Spain, water from alcoholic beverages by men from Italy, and also water from hot drinks from French men and women. Spain and France were the highest consumers of alcoholic beverages, especially by men, although consumption was similar in the three countries. Spain was also the country with the lowest consumption of water from foods. Regarding soft drinks, Italy had a lower water intake from both diet and caloric beverages.

**Figure 2:**
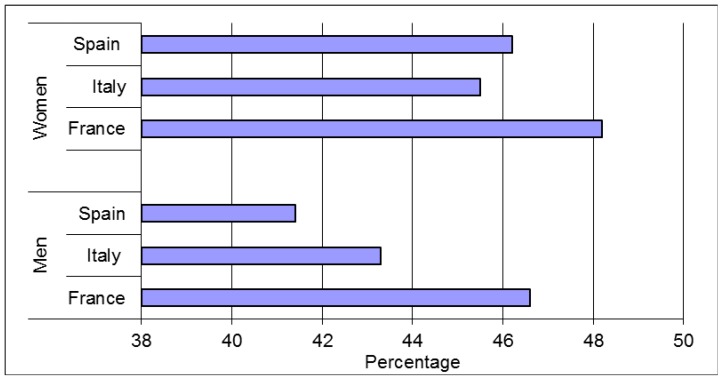
Percentage of water consumption in the beverage category by country and gender.

Figure 2 represented the percentage of water consumption as a beverage category on average over the three day study period. It was represented separately from the others’ beverages consumed in order to clearly highlight the differences in intake by country. The percentage of water consumption was 48% for women and 47% for men in France—the highest percentage of the three countries studied. In Spain and Italy, the higher percentage was for Spain in the female group, and was less for Italian males.

**Figure 3:**
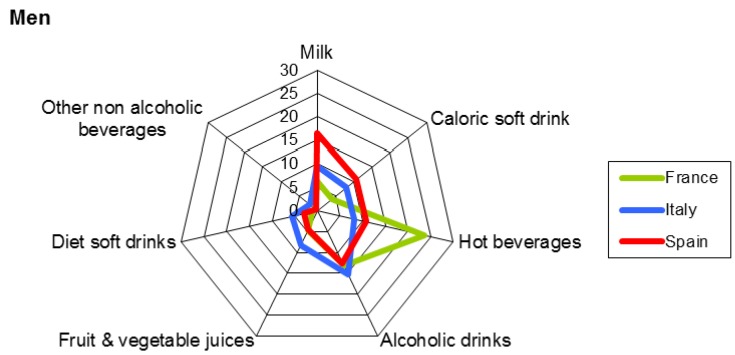
Percentage of each category of beverages consumed on average over the assessment period by men (18–75 years). Water as a beverage is not included in this figure.

**Figure 4:**
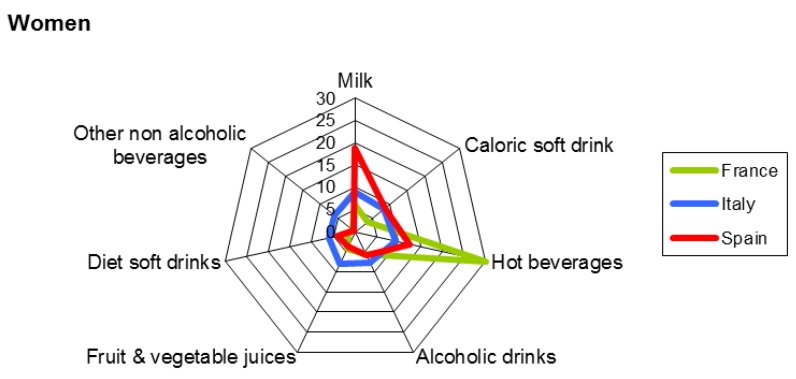
Percentage of each category of beverages consumed on average over the assessment period by women (18–75 years). Water as a beverage is not included in this figure.

The percentages of each beverage category consumed over the assessment period by gender are presented in Figure 3 and Figure 4. Water as a beverage category is not included in them. On average, among men, hot beverages were the most frequently consumed beverages in France, followed by milk in Spain and alcoholic drinks in Italy, with percentages of 23%, 17%, and 15%, respectively. For women, the list in decreasing order was hot beverages in France (30%) and milk in Spain (19%). For Italy, the highest percentage was also for hot beverages (9%).

Appendix Table A1 shows the percentiles of beverage consumption (gr/day) of each country among adults by sex. For both men and women, the main source was water for the three countries. Spain consumed higher amounts of milk, followed by hot beverages and alcoholic drinks for women and in the opposite order (alcoholic drinks—hot beverages) by men. For Italy, the highest values were hot drinks for both men and women, followed by alcoholic drinks for men. For France, the second-most consumed beverage was hot beverages, followed by milk for women and alcohol for men.

Participants who fulfilled the EFSA AI recommendations for TWI for men and women (2.5 L and 2.0 L, respectively) were classified under Criterion 1. Participants with ratios of water/EI >1.0 were included under Criterion 2 (considering a value of 1 g of water per 1 kcal of EI). Finally, participants who met both definitions (Criterias 1 and 2) were classified under Criterion 3. Following this analysis, Table 3 shows that the group with the highest percentage of compliance with the criteria was women in France (51% fulfilled Criterion 1, 66% Criterion 2, and 46% Criterion 3). In the case of French men, no criteria exceeded 50% compliance (30% met Criterion 1, 46% Criterion 2, and 24% Criterion 3). Furthermore, Criterion 2 obtained the highest percentage of compliance obtained among the three countries. However, it is very noticeable that women achieved higher rates of compliance than men in all criteria and in all countries included in the analyses.

## 4. Discussion

This study addresses the analysis of beverage intake in adults from three national European databases from France, Italy, and Spain. Fluid intake was examined using a similar methodology and the same data classification method in the three countries.

The analyses indicate that only women from France complied with the recommended value of 2.1 L (SE 2.4). Spain had the lowest water intake among the three, with 1.7 L (SE 22.9) for men and 1.6 L (SE 19.4) for women. It is evident that the insufficient consumption of water according to EFSA reference values and the high percentage of adults who did not comply with the recommendation requires more attention—not only from the scientific community, but also from the public health and education communities.

France met EFSA recommendations most closely. Similar situations have happened when the criterion applied was the ratio between TWI and total EI. However, with this same criterion, the results appear to show better compliance for the three countries. Between 50% and 80% of women and 70%–85% of men did not meet EFSA recommendations for water consumption. As we expected, TWI differed by gender in the three countries, and was always higher for women. This leads one to consider that women may tend to have healthier lifestyle patterns than men [28], or it is also possible that men are more likely to underreport fluids than women [29].

Regarding EI, the three countries recorded EIs from beverages of 12%, 8%, and 6% for Spain, France, and Italy, respectively. These percentages are very close to the 10% proposed by some international authorities [13,30], who recommended that no more than 10% of daily calorie intake should come from beverages. It is to be noted that dietary patterns are crucial in determining the percentage of calories provided by beverages. In our study, the lower percentage (6%) is derived from the higher EI (2137.9 kcal/day in Italy). In this case, although climate and cultural conditions are similar, the beverage consumption level is different; French people have a higher water intake than the others from all sources, and this can be related to a lower EI according to the major sources highlighted in Figure 1.

The compilation of the three national surveys, conducted with large samples of participants, which also kept the same classification of the beverages, is one of the most important strengths of this study. It demonstrates that water constitutes the largest proportion of total fluid intake for the three countries examined. The data of the current analysis are in line with data reported in other previously-published surveys [31].

The fact remains that there is little information regarding beverage consumption in the EU and even less that describes patterns of habitual intake. Fluid intake is part of our eating habits; it is influenced by climate and by our physiological needs, as well as by our customs. The amount of liquids consumed and our drinking patterns have consequences on our health status. Although the purpose of this study was to describe the usual profiles of water consumption and drinks in the EU, and to analyze similarities and differences in the consumption profiles of the populations of the regions studied, we are well aware that three studies are not sufficient to achieve the illustration of the status of actual water intake in Europe, nor does it reflect the possible consequences on health; this is the major limitation. However, it is enough to show the existing trend of low consumption, which is much more evident in men than it is in women and in older populations. This point deserves special attention. One important reason for this is the fact that the proportion of elderly people in the population has been greatly increasing around the world over the last decade, and these trends are expected to continue [32]. Europe is not unaware of this situation. Since the 1970s, when there were about 3.3 million people over the age of 65, this number has increased to 7.6 million in Europe, which accounts for more than 17.1% of the population. The elderly population has a high rate of chronic illness [33], and is more vulnerable to disease. The evidence of dehydration among this group is well known and documented [34,35]. Dehydration substantially increases the burden of health care in a direct way, as a disease itself, or indirectly as a comorbidity of other diseases. Therefore, this represents an important public health issue by imposing a significant economic burden [36].

On the other hand, for nutritional epidemiology, the focus of interest is based on estimating the risk of inadequate beverage intake. Methods of estimating beverage adequacy have changed over time. We have gone from estimating it from questionnaires or surveys of general food and beverages (which do not focus attention on beverage intake), to the exclusive use of questionnaires collecting information on beverage intake, which have been validated with appropriate biomarkers. However, the complexity and costs underlying the evaluation of beverage consumption do not suffice, in view of the few exclusive beverage studies that exist in the EU [37]. Current epidemiological studies that focus on beverage intake have large differences in the values obtained. A significant part of these differences are derived from the methodology used. The choice of an appropriate method is essential in order to established a precise and reliable record. The use of different methodologies leads to obtaining such disparate results that they are impossible to compare [2]. Standardization of procedures for collecting data from different studies is the first and most important step to take when such studies arise. Our experience suggests that a comparison of different methods results in a higher water intake when the food frequency questionnaires (FFQ) method for fluid intake is used [38]. Multiple-day (three or seven days) food records and 24-h dietary recalls have been used successfully [39,40]. These seem to improve the assessment of water throughout the day, and can be very useful in nutrition surveys. In fact, in this analysis, we have been forced to discard some studies that could potentially have been included in the current work due to the high differences in the results caused by the use of different methodologies.

The interpretation of these results should be carefully considered for a number of reasons. First, the number of studies that were eligible for inclusion in this analysis was small, which limited the possibility of showing the real pattern of fluid consumption in Europe. Furthermore, although the statistical analysis was the same for the three countries included, the difference in the recruitment and sampling of the studies could explain some of the obtained results. For example, the higher level of University Education found in the French population [8,26] was because the sample included mostly volunteers; that is, more educated people for research [41]. Second, the studies were cross-sectional in design, which provides evidence for associations, but not causal relationships. Furthermore, it is well-known that intake assessed by self-reports is subject to random and systematic reporting errors, which may introduce bias into the estimate of water consumption. However, according to the scientific literature [42], these kinds of records continue to be the chosen dietary assessment methods for epidemiological studies.

## 5. Conclusions

This study attempts to show actual water consumption in some EU countries. It highlights the need to formulate appropriate health and nutrition policies in order to increase TWI in these populations.

Without a doubt, Europe needs a collective effort to contribute to standardizing the assessment of beverage intakes, as well as to minimize the use of inappropriate dietary instruments. The future of beverage intake assessment requires the use of new instruments and techniques, the application of available new technologies, and an open discussion regarding the clarity and transparency of the procedures required.

## Figures and Tables

**Table 1:** Demographic and anthropometric details for included study participants from Spain (ANIBES dataset), France (NutriNet-Santé dataset) and Italy (INRAN-SCAI dataset).

		*n*	SPAIN			FRANCE			ITALY			p*Value		
			Men	Women	Total	Men	Women	Total	Men	Women	Total	Men	Women	Total
			895	964	1859	20,636	74303	94,939	1202	1245	2313	-	-	-
Age Group	18–64 (years)	%	89.5	88.7	88.9	87.17	95.38	93.59	88.9	88.6	88.7	0.643	0.006	0.007
	65–75 (years)	%	10.5	11.3	11.1	12.83	4.62	6.41	11.1	11.4	11.3	0.047	<0.001	<0.001
Level of education	Primary	%	26.4	27.5	27.0	23.14	17.70	18.88	34.2	37.4	35.9	<0.001	<0.001	<0.001
	Secondary	%	49.6	47.9	48.7	13.67	18.44	17.40	44.6	40.2	42.2	<0.001	<0.001	<0.001
	University	%	24.0	24.7	24.3	63.20	63.87	63.72	21.2	22.4	21.9	<0.001	<0.001	<0.001
Rate of unemployment		%	18.2	8.6	13.5	4.96	6.47	6.14	2.4	2.7	2.6	<0.001	<0.001	<0.001
Weight (kg)		Mean (SE)	82.4 (0.56)	66.8 (0.48)	74.4 (0.42)	77.4 (0.09)	63.3 (0.05)	66.4 (0.05)	78.4 (0.3)	62.7 (0.3)	69.9 (0.3)	<0.001	<0.001	<0.001
Height (cm)		Mean (SE)	174.5 (0.25)	161.2 (0.22)	167.7 (0.24)	176.5 (0.05)	164.1 (0.02)	166.8 (0.03)	175.0 (0.2)	163.0 (0.2)	169.0 (0.2)	<0.001	<0.001	<0.001
BMI (kg/m^2^)		Mean (SE)	27.1 (0.18)	25.7 (0.19)	26.4 (0.13)	24.8 (0.03)	23.5 (0.02)	23.8 (0.01)	26.0 (0.1)	23.0 (0.1)	24.0 (0.1)	<0.001	<0.001	<0.001
Waist Circumference (cm)		Mean (SE)	94.0 (0.49)	83.1 (0.46)	88.4 (0.36)	90.3 (0.16)	79.9 (0.11)	82.8 (0.10)	Unrecorded	Unrecorded	Unrecorded	<0.001	<0.001	<0.001
BMI class	Healthy weight	%	36.5	47.5	42.2	58.8	72.6	69.6	50.3	71.3	61.6	<0.001	<0.001	<0.001
	Overweight	%	40.0	32.0	35.9	32.3	18.3	21.4	39.4	21.9	30.0	<0.001	<0.001	<0.001
	Obese	%	22.9	17.8	20.3	8.8	9.1	9.0	10.2	6.8	8.4	<0.001	<0.001	<0.001

p*value obtained through chi-squared test and ANOVA-test = significant (<0.001). BMI: body mass index.

**Table 2:** Contribution of food and beverages to total water and energy intake by gender among the populations included.

	SPAIN						FRANCE						ITALY					
	Contribution to Water Intake (g/Day)			Contribution to Energy Intake (kcal/Day)			Contribution to Water Intake (g/Day)			Contribution to Energy Intake (kcal/Day)			Contribution to Water Intake (g/Day)			Contribution to Energy Intake (kcal/Day)		
	Women	Men	Total	Women	Men	Total	Women	Men	Total	Women	Men	Total	Women	Men	Total	Women	Men	Total
All food & beverages	1605.2	1702.9	1652.3	1653.3	1939,1	1790.8	2101.7	2251.0	2134.2	1783.0	2250.1	1884.5	1667.9	1768.6	1714.3	1929.6	2381.4	2137.9
Mean (SE)	(19.4)	(22.9)	(14.9)	(13.9)	(17.6)	(11.6)	(2.4)	(4.7)	(2.2)	(1.5)	(3.6)	(1.5)	(14.1)	(16.9)	(10.9)	(14)	(18.5)	(12.2)
Food only %	31.99	32.54	32.25	88.49	87.20	87.87	37.8	39.4	38.1	92.2	90.1	91.7	42.2	44.4	44.8	95.3	93.4	94.4
Beverages only %	68.61	67.46	67.75	11.51	12.80	12.13	62.2	60.6	61.9	7.8	9.9	8.3	55.8	55.5	55.7	4.7	6.6	5.6

**Table 3:** Combined classification for Total Water Intake (TWI) following the established criteria.

EFSA 2.5 L Men; 2.0 L Women	Men			Women		
	Spain	Italy	France	Spain	Italy	France
*n*	865	1202	20636	964	1245	74,303
Criterion 1: *n* (%)	114 (13)	127 (11)	6281 (30)	216 (22)	336 (24)	38,152 (51)
Criterion 2: *n* (%)	262 (29)	160 (13)	9446 (46)	404 (42)	379 (27)	49,135 (66)
Criterion 3 (1 and 2): *n* (%)	98 (11)	66 (5)	5029 (24)	185 (19)	210 (15)	34,065 (46)

EFSA: European Food Safety Authority [13]: Criterion 1: TWI >2.5 L men, >2 L women (aged 14 to 75 years); Criterion 2: Ratio of total water/total energy intakes >1; Criterion 3: Both criteria.

## Appendix A

**Table A1:** Percentiles of beverage consumption (g/day) among adults by sex.

Adults 18–75 Years	Spain												Italy												France											
	Total				Women				Men				Total				Women				Men				Total				Women				Men			
	*n*	P 25	P 50	P 75	*n*	P 25	P 50	P 75	*n*	P 25	P 50	P 75	*n*	P 25	P 50	P 75	*n*	P 25	P 50	P 75	*n*	P 25	P 50	P 75	*n*	P 25	P 50	P 75	*n*	P 25	P 50	P 75	*n*	P 25	P 50	P 75
Hot bev. (g/day)	1655	33.3	100.0	183.3	857	46.7	108.3	193.3	798	26.7	90.0	171.7	2313	60.0	113.6	180.0	1245	60.0	115.0	180.0	1068	60.0	110.0	180.0	94,939	150.0	339.3	567.8	74,303	160.7	353.6	594.6	20,636	116.4	294.4	475.0
Milk (g/day)	1655	90.0	173.3	266.7	857	101.7	183.3	273.3	798	70.0	155.0	258.3	2313	0.0	0.0	50.0	1245	0.0	0.0	83.3	1068	0.0	0.0	0.0	94,939	0.0	0.0	123.8	74,303	0.0	0.0	120.0	20,636	0.0	0.0	139.3
Juices (g/day)	1655	0.0	0.0	66.7	857	0.0	0.0	66.7	798	0.0	0.0	66.7	2313	0.0	0.0	2.0	1245	0.0	0.0	2.0	1068	0.0	0.0	2.0	94,939	0.0	0.0	96.4	74,303	0.0	0.0	90.8	20,636	0.0	0.0	120.0
Caloric soft bev. (g/day)	1655	0.0	0.0	133.3	857	0.0	0.0	110.0	798	0.0	0.0	166.7	2313	0.0	0.0	0.0	1245	0.0	0.0	0.0	1068	0.0	0.0	0.0	94,939	0.0	0.0	42.8	74,303	0.0	0.0	42.9	20,636	0.0	0.0	53.6
Diet soft bev.(g/day)	1655	0.0	0.0	0.0	857	0.0	0.0	0.0	798	0.0	0.0	0.0	2313	0.0	0.0	0.0	1245	0.0	0.0	0.0	1068	0.0	0.0	0.0	94,939	0.0	0.0	0.0	74,303	0.0	0.0	0.0	20,636	0.0	0.0	0.0
Alcohol (g/day)	1655	0.0	0.0	140.0	857	0.0	0.0	85.0	798	0.0	44.2	220.0	2313	0.0	0.0	0.0	1245	0.0	0.0	93.3	1068	0.0	110.0	255.7	94,939	0.0	35.7	132.2	74,303	0.0	21.6	100.4	20,636	0.0	112.2	265.7
Water (g/day)	1655	200.0	450.0	863.3	857	250.0	456.7	873.3	798	166.7	450.0	850.0	2313	373.3	586.7	853.3	1245	400.0	600.0	853.3	1068	320.0	586.7	826.7	94,939	354.3	591.4	907.2	74,303	354.3	589.3	900.0	20,636	353.6	604.3	932.2
Other non-alcohol bev. (g/day)	1655	0.0	0.0	0.0	857	0.0	0.0	0.0	798	0.0	0.0	0.0	2313	0.0	0.0	0.0	1245	0.0	0.0	0.0	1068	0.0	0.0	0.0	94,939	0.0	0.0	0.0	74,303	0.0	0.0	0.0	20,636	0.0	0.0	0.0
